# The impact of pre-rehydration guided by carotid corrected flow time on hypotension prevention following general anesthesia induction in patients undergoing gastrointestinal surgery: a prospective randomized controlled trial

**DOI:** 10.3389/fmed.2024.1416574

**Published:** 2024-05-31

**Authors:** Min Li, Feng Li, Jiali Yu, Xixi Tang, Chengfu Zhou, Qi Chen, Hongliang Liu

**Affiliations:** ^1^Department of Anesthesiology, Chongqing University Cancer Hospital, Chongqing, China; ^2^Department of Anesthesiology, Zibo First Hospital, Zibo, China

**Keywords:** carotid corrected flow time, hypotension, general anesthesia, pre-rehydration, gastrointestinal surgery

## Abstract

**Background:**

Patients undergoing gastrointestinal surgery often experience hypotension following general anesthesia induction due to insufficient volume. This study aimed to assess whether pre-rehydration guided by carotid corrected flow time (FTc) could mitigate post-induction hypotension induced by general anesthesia.

**Methods:**

Patients undergoing resection of gastrointestinal tumors were assigned to either the conventional treatment group (Group C) or the fluid treatment group based on FTc (Group F). Within Group F, patients were further divided into Group A (carotid FTc <340.7 ms) and Group B (carotid FTc ≥340.7 ms) based on pre-rehydration carotid FTc values. Group A patients received pre-rehydration with 250 mL of colloids (hydroxyethyl starch—HES) administered within 15 min until carotid FTc reached ≥340.7 ms to counteract hypovolemia prior to induction. Patients in Group B and Group C received a continuous HES infusion at a rate of 6 mL/kg/h 30 min before induction to compensate for physiological fluid loss. All patients received a perioperative background infusion of 3 mL/kg/h compound sodium chloride, with infusion rates optimized based on mean arterial pressure (MAP) and heart rate (HR). The incidence of post-induction hypotension was compared between Group C and Group F, as well as between Group A and Group B.

**Results:**

The incidence of hypotension after induction was significantly lower in Group F compared to Group C (26.4% vs. 46.7%, respectively; *p* < 0.001). Patients in Group A received significantly more pre-rehydration, leading to a greater increase in carotid FTc values compared to Group B (336.5 ± 64.5 vs. 174.3 ± 34.1 ms, *p* = 0.002). However, no significant difference in carotid FTc values after pre-rehydration was observed between the groups. There was no significant difference in the incidence of hypotension after general anesthesia induction between Group A and Group B (22.9% vs. 28.8%, *p* = 0.535).

**Conclusion:**

Pre-rehydration based on FTc can effectively reduce the occurrence of post-induction hypotension in patients undergoing gastrointestinal surgery who present with insufficient volume.

**Clinical trial registration:**

https://www.chictr.org.cn/showprojEN.html?proj=201481.

## 1 Introduction

It can be challenging to accurately assess blood volume and fluid responsiveness in patients with gastrointestinal tumors, who often undergo prolonged fasting or intestinal preparation before surgery (1, 2). Consequently, they are more susceptible to experiencing hypotension after general anesthesia induction. While dynamic indicators like pulse pressure variation (PPV) and stroke volume variation (SVV) are excellent predictors of fluid responsiveness (3–6), their reliability is limited to mechanically ventilated patients (7, 8). Carotid corrected flow time (FTc) changes offer a technically simple, reliable, and non-invasive method for predicting blood volume without requiring arterial cannulation. Several studies have demonstrated that carotid artery FTc remains unaffected by respiration and can effectively predict blood volume in spontaneously breathing patients (9–13). For instance, Chen et al. (13) identified an optimal cutoff value for FTc of 340.7 ms, with areas under the receiver operating characteristic curves (AUROCs) of 0.811, a sensitivity of 76.8% and specificity of 80.5% for predicting induced hypotension.

This study aims to investigate whether pre-rehydration guided by a FTc cutoff value of <340.7 ms could reduce the occurrence of hypotension induced by general anesthesia in patients undergoing gastrointestinal surgery.

## 2 Methods

### 2.1 Study population

Following approval by the Medical Ethics Committee of the Cancer Hospital Affiliated with Chongqing University, this study was registered in the ClinicalTrials Research Registry (ChiCTR2300075547). Patients undergoing gastrointestinal tumor resection at our hospital between October 2023 and February 2024 were enrolled and randomly assigned to either the conventional treatment group (Group C) or the fluid treatment group based on FTc (Group F) using a random number table method. Within Group F, patients were further divided into Group A (carotid FTc <340.7 ms) and Group B (carotid FTc ≥340.7 ms) based on pre-rehydration carotid FTc values. Patients meeting any of the following criteria were excluded from the study: (1) History of neck trauma or surgery. (2) Carotid atherosclerosis causes more than 1/3 of lumen stenosis or abnormal neck vascular anatomy. (3) American Society of Anesthesiologists classification >III. (4) Body mass index >35 kg/m^2^ or <16 kg/m^2^. (5) Presence of rhythm disorders, cardioverters, or implantable pacemakers. (6) Presence of severe heart diseases, such as history of cardiomyopathy or valvular heart disease. Signed informed consent was obtained from all participants or their families.

### 2.2 Study procedure

All patients underwent an 8 h fast for solids and a 4 h fast for liquids. Additionally, they received a coloclyster until achieving a satisfactory level of intestinal sanitary conditions before the operation. Upon arrival in the operating room, patients’ electrocardiogram (ECG), heart rate (HR), and pulse oxygen saturation (SpO2) were monitored. Radial artery catheterization was performed under local anesthesia to enable continuous monitoring of arterial blood pressure. Carotid ultrasound was employed to measure carotid FTc values. In Group A, patients received pre-rehydration with 250 mL of colloids (hydroxyethyl starch—HES) each time until carotid FTc reached ≥340.7 ms to offset hypovolemia before induction, within a 15 min timeframe (14). Conversely, patients in Group C and Group B received a continuous infusion of HES at a rate of 6 mL/kg/h for 30 min before induction to compensate for physiological fluid loss (15). Anesthesia induction: propofol (2 mg/kg), sufentanil (0.3 μg/kg), and tracheal intubation was facilitated after 3 min of intravenous cisatracurium (0.15 mg/kg) administration. The lowest recorded values of systolic blood pressure (SBP), diastolic blood pressure (DBP), mean arterial pressure (MAP), and HR after induction until successful intubation were documented by non-invasive continuous blood pressure and electrocardiogram monitoring. Hypotension was defined as an absolute MAP below 65 mmHg or a decrease of greater than 20% from baseline MAP after the induction of general anesthesia (16). Subsequently, all patients received a perioperative background infusion volume of 3 mL/kg/h compound sodium chloride. The infusion rate was adjusted based on MAP and HR parameters.

### 2.3 Methods for ultrasound measurement of carotid FTc

A portable ultrasound device (Mindray Medical Systems, Shenzhen, China) was utilized for ultrasound examinations and measurements conducted by an experienced sonographer. The procedure for obtaining images of the common carotid artery was as follows: (1) The patient was positioned supine, with their head rotated 30° to the left. (2) The high-frequency linear array transducer was placed transversely at the lower border of the thyroid cartilage, with the probe marker directed towards the left, ensuring the common carotid artery was centered on the screen. (3) The probe was rotated counterclockwise to obtain a long-axis B-mode image of the common carotid artery, approximately 2 cm proximal to the carotid bifurcation. (4) The sample volume was placed at the center of the arterial vessel, and the cursor angle was adjusted parallel to the direction of blood flow, with an angle of ≤60°. Optimal sampling volume and angle were adjusted for a satisfactory spectrum and then frozen. Subsequently, the caliper function on the machine was used to measure the parameters. (5) The FTc value was measured from the start of the systolic upstroke to the dicrotic notch. Heart rate (HR) was automatically calculated from the measurement intervals between the beginning of two consecutive Doppler flow upstrokes. The average of three consecutive cycles was taken. (6) FTc was calculated to compensate for HR using the Wodey formula: FTc = FT + [1.29 × (HR − 60)] (10).

**Figure 1:**
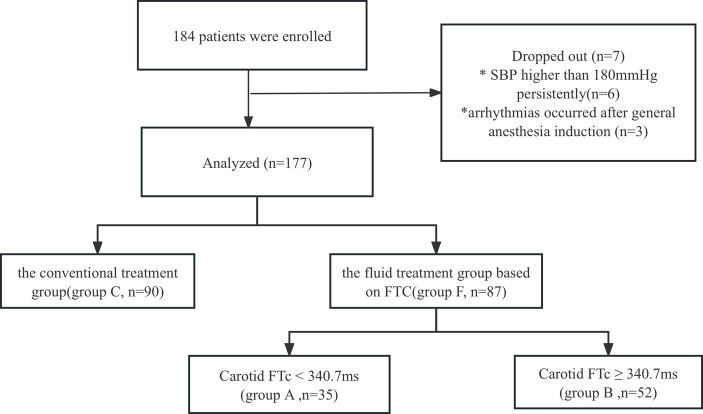
The incidence of hypotension after general anesthesia induction between Group C and Group F.

### 2.4 Sample size calculation and statistical analysis

Statistical analysis was conducted using SPSS version 26.0. Measurement data were presented as mean ± standard deviation, Comparisons between groups for age, BMI, MAP, FTC, and pre-rehydration volume were conducted using student’s *t*-test for normally distributed data and the Mann–Whitney test for non-normally distributed data. Categorical variables were presented as percentages, and the chi-square test was used for ASA grade, hypertension, and the incidence of hypotension after general anesthesia induction when the theoretical frequency was ≥5. Fisher’s exact test was employed for frequencies less than 5. A significance level of *p* < 0.05 was considered statistically significant.

Based on previous relevant statistical data, the incidence of hypotension after induction was 67.7%, while our pre-test showed an incidence of 42.8% (2). The sample size for this trial was calculated using PASS 15.0 software to achieve a power of 0.9 and an alpha of 0.15 test. Considering a 10% allowance for potential dropouts, a total of 92 patients were required in both Group C and Group F.

## 3 Results

A total of 184 patients undergoing gastrointestinal tumor resection were initially allocated into Group C and Group F. However, 6 patients were excluded from the study due to persistently elevated systolic blood pressure (SBP) higher than 180 mmHg, and 1 patient was excluded due to post-general anesthesia induction arrhythmias. Consequently, the final analysis included 177 patients (Group C, *n* = 90; Group F, *n* = 87) (refer to Figure 1). There were no statistically significant differences in demographic characteristics between Group C and Group F (*p* > 0.05) (Table 1).

**Table 1:** Comparison of patients’ demographic characteristics between Group C and Group F.

	Group C (*n* = 90)	Group F (*n* = 87)	*p*-value
Male/female	56/34	54/33	0.983
Age (y)	63.0 ± 8.8	61.0 ± 8.4	0.440
BMI (kg/m^2^)	22.3 ± 2.1	24.0 ± 2.5	0.205
ASA (II/III)	78/12	71/16	0.357
Hypertension	23 (25.6%)	18 (20.7%)	0.443
Baseline SBP (mmHg)	133.0 ± 17.1	140.8 ± 16.8	0.618
Baseline DBP (mmHg)	72.4 ± 9.7	74.5 ± 10.1	0.309
Baseline MAP (mmHg)	85.1 ± 9.0	96.7 ± 11.1	0.121
Baseline HR (bpm)	72.9 ± 7.2	71.1 ± 8.5	0.145

BMI, body mass index; ASA, American Society of Anesthesiologists physical status; SBP, systolic blood pressure; DBP, diastolic blood pressure; MAP, mean arterial pressure; HR, heart rate. Data are presented as mean (standard deviation), number of patients (%).

**Figure 2:**
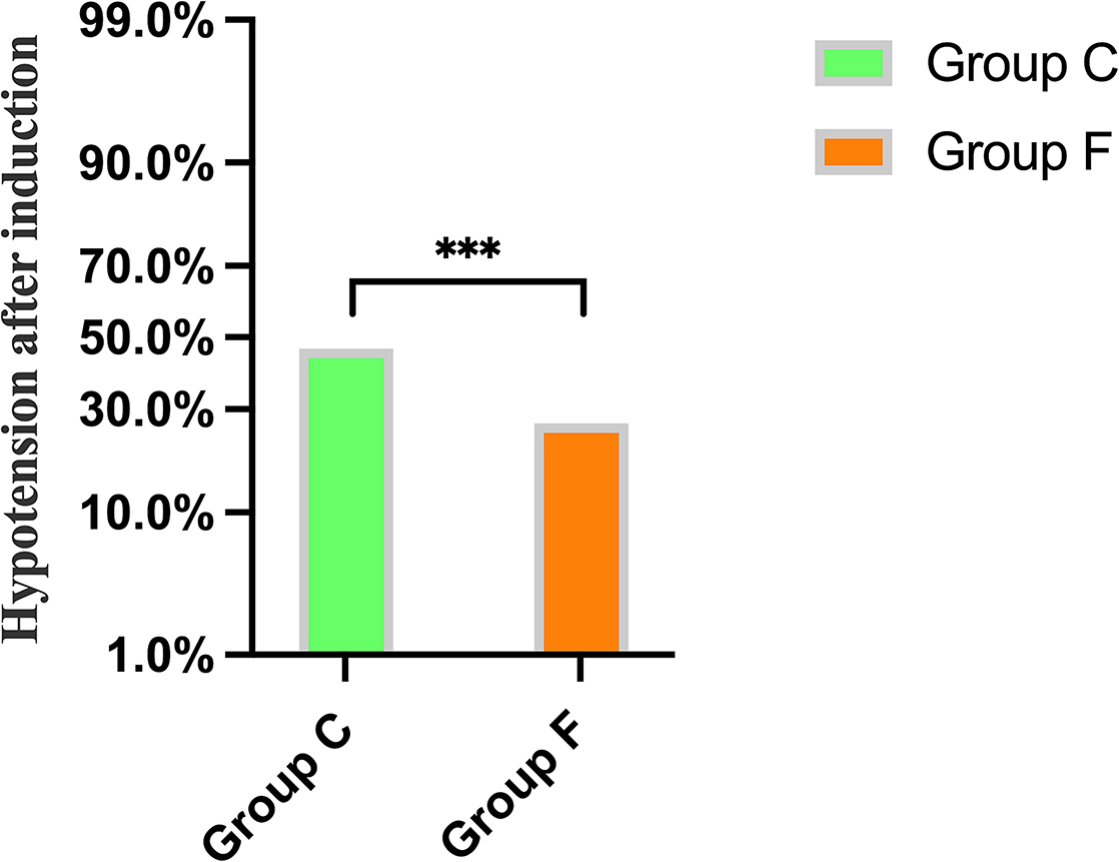
Comparison of the incidence of hypotension after general anesthesia induction between Group C and Group F. ^***^p < 0.001 showed significant difference.

A significant difference was observed between Group C and Group F regarding the incidence of hypotension after general anesthesia induction, with rates of 46.7 and 26.4%, respectively (*p* < 0.001) (Figure 2).

**Figure 3:**
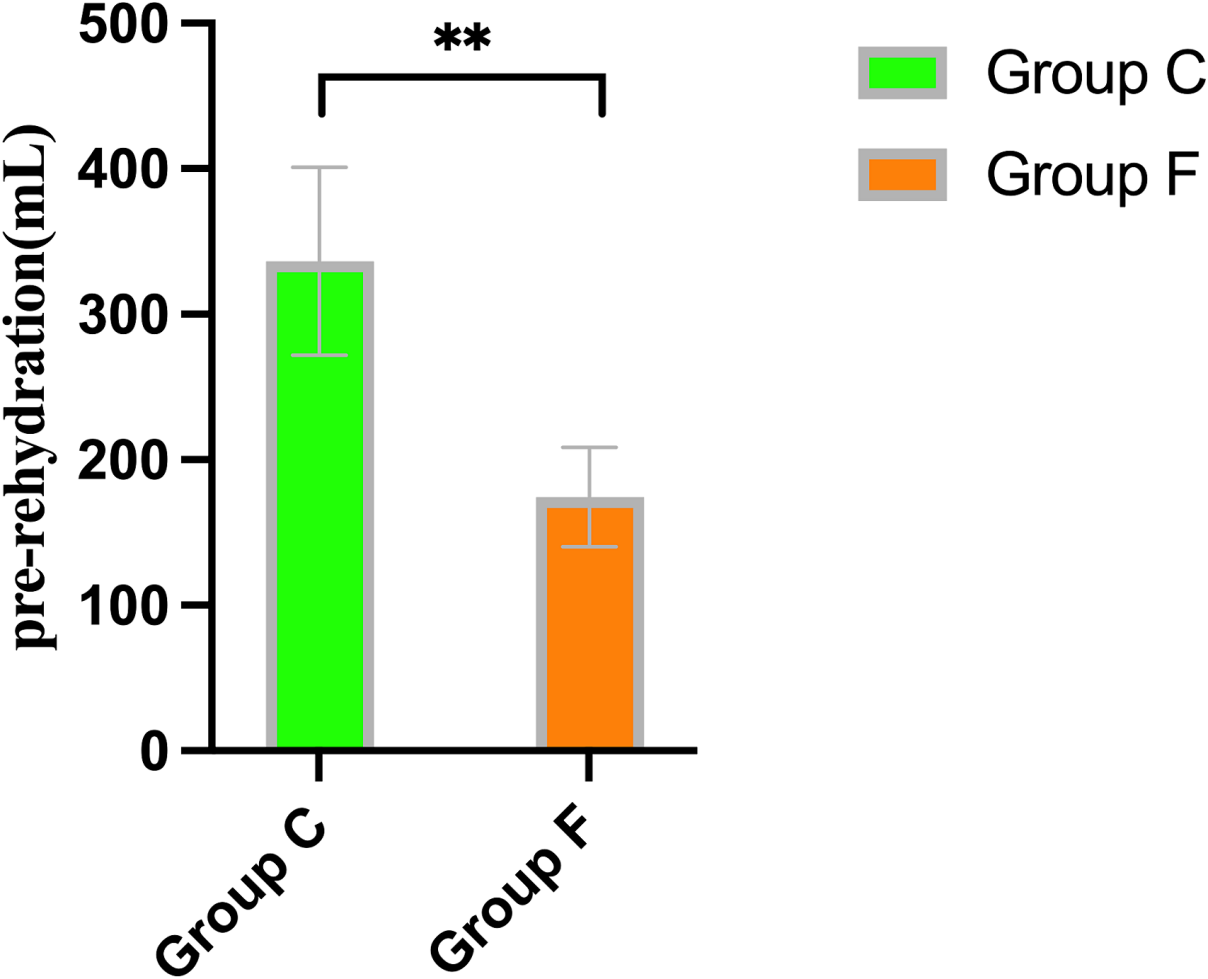
Comparison of the volume of pre-rehydration before induction between Group A and Group B. ^**^p < 0.01 showed significant difference.

Patients in Group A received significantly more pre-rehydration, leading to a greater increase in carotid FTc values compared to patients in Group B (*p* = 0.002) (Figure 3). However, there were no discernible differences between the groups in carotid FTc values after pre-rehydration (*p* > 0.05) (Table 2).

**Table 2:** Comparison of carotid FTc before and after rehydration between Group A and Group B.

	Group A	Group B	*p*-value
Before rehydration (ms)	324.5 ± 7.0	363.0 ± 11.1	0.038^*^
After rehydration (ms)	351.9 ± 8.1	374.6 ± 11.5	0.073

Data are mean ± standard deviation.^*^p < 0.05 showed statistical difference.

**Figure 4:**
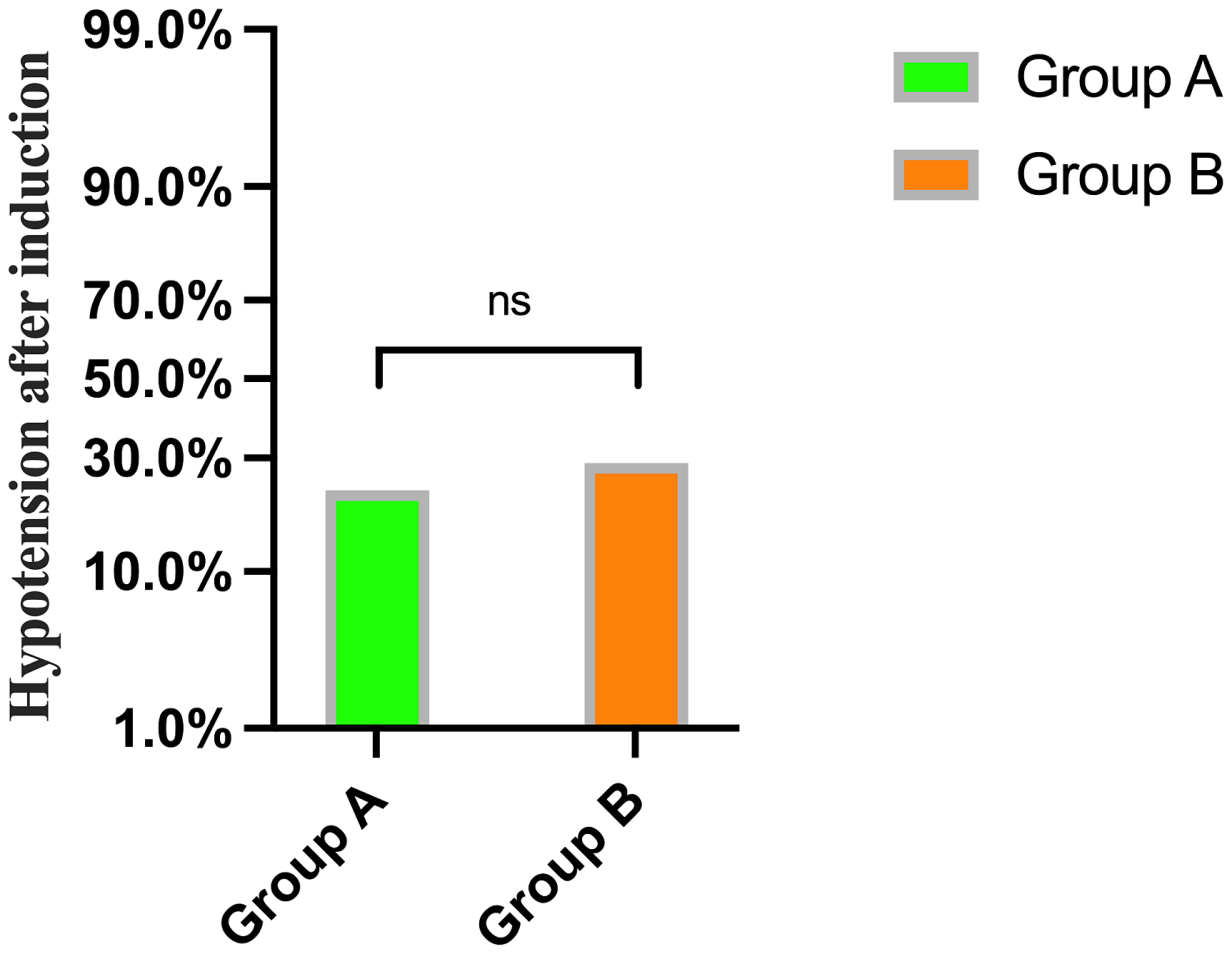
The incidence of hypotension after general anesthesia induction between Group A and Group B.

No significant differences were observed in mean arterial pressure (MAP) and heart rate (HR) before and after rehydration and after induction (*p* > 0.05) (Table 3). Additionally, there was no significant difference in the incidence of hypotension after induction of general anesthesia between the two groups (*p* = 0.535) (Figure 4).

**Table 3:** Changes of MAP and HR before and after rehydration, and after induction between Group A and Group B.

		Group A	Group B	*p*-value
MAP (mmHg)	Before rehydration	99.5 ± 9.8	94.8 ± 11.7	0.360
	After rehydration	99.8 ± 10.1	95.2 ± 11.0	0.868
	After induction	76.4 ± 11.5	74.9 ± 11.8	0.961
HR (bpm)	Before rehydration	71.9 ± 9.0	71.4 ± 7.1	0.143
	After rehydration	70.7 ± 8.2	71.8 ± 6.3	0.061
	After induction	65.5 ± 6.6	64.0 ± 5.2	0.168

MAP, mean arterial pressure; HR, heart rate. Data are mean ± standard deviation.

## 4 Discussion

In this trial, the incidence of hypotension in the conventional treatment group and the fluid treatment group based on FTc was 46.7 and 26.4%, respectively. Following pre-rehydration with a cutoff value of FTc <340.7 ms, the incidence of hypotension after general anesthesia induction between groups was 22.9 and 28.8%, respectively. This suggests that pre-rehydration based on FTc may be an effective method to reduce the occurrence of hypotension induced by general anesthesia in patients with gastrointestinal tumors.

Patients with gastrointestinal tumors are more susceptible to insufficient blood volume and subsequent post-induction hypotension due to the disease itself and prolonged intestinal preparation (2). Intraoperative hypotension is associated with delayed awakening, acute kidney injury, myocardial ischemia, and increased perioperative mortality (17, 18). Therefore, accurately assessing a patient’s volume status and implementing appropriate therapeutic measures are crucial. No significant differences were found in general condition and clinical data between the groups, indicating well-balanced preoperative conditions. There were also no significant statistical differences in anesthesia methods between the groups, suggesting similar physiological effects of anesthesia. Consequently, the two groups were overall comparable. The measurement of FTc using ultrasonography in the carotid artery as a new non-invasive volumetric state measurement method was initially introduced by Blehar et al. (19). This method is based on the rationale that FTc in the descending thoracic aorta measured by esophageal Doppler has been shown to be helpful for volume optimization. In their preliminary study, they observed that after a mean fluid resuscitation of 1,110 mL in dehydrated patients, FTc increased from a pre-fluid mean of 299 ms to a post-fluid mean of 340 ms. Additionally, Hossein-Nejad reported that FTc significantly decreased from a mean of 345.07 ± 37.19 ms to 307.77 ± 31.76 ms after hemodialysis in patients with end-stage renal disease, who are generally considered to be hypervolemic (20). These findings suggest that FTc may reliably reflect blood volume status.

In this trial, the incidence of hypotension in the conventional treatment group and the fluid treatment group based on FTc was 46.7 and 26.4%, respectively, indicating that fluid treatment based on FTc can reduce the incidence of hypotension induced by general anesthesia induction in patients undergoing gastrointestinal tumor surgery. In recent years, multiple studies have shown that FTc is a reasonable predictive indicator of hypotension induced by general anesthesia (2, 10, 13, 21). According to different surgical types and populations, the FTc cutoff values range from 330.2 ms to 349.4 ms when hypotension occurs after general anesthesia. Chen et al. (13) reported the optimal cutoff value for capacity reactivity of FTc was 340.74 ms in patients who underwent abdominal surgery under general anesthesia. These patients underwent preoperative bowel preparation, which is closest to our experimental population. Therefore, we chose 340.7 ms as the optimal cutoff value for volume responsiveness, which means the carotid FTc <340.7 ms before anesthesia induction mirrors the patient’s blood volume was insufficient and were prone to suffer from post-induction hypotension. Currently, there are few reports on whether the combination of carotid artery FTc and pre-rehydration can reduce the incidence of hypotension induced by general anesthesia in patients with gastrointestinal tumors.

In this trial, the FTc of Group A before anesthesia induction was smaller than that of Group B and less than 340.7 ms, indicating that patients in Group A were in a state of insufficient blood volume before induction. Group A experienced an increase in FTc values after rehydration, attributed to the amount of rehydration received. No discernible disparity was observed in FTc between the two groups after rehydration. Both groups experienced decreases in heart rate (HR) and mean arterial pressure (MAP) after induction. The primary factors contributing to this phenomenon include the vasodilatory effects of anesthetics, cardiovascular depression, and the patient’s volume status. However, HR, MAP, and the incidence of hypotension were similar between the two groups, indicating that a certain amount of pre-rehydration in patients with insufficient volume before induction can effectively reduce the occurrence of hypotension during the perioperative period. A recent study reported that elderly patients with an FTc <334.95 ms after receiving 8 mL/kg of pre-rehydration experienced a reduction in hypotension induced by general anesthesia, aligning with our findings. However, the reduction in hypotension incidence decreased from 92.31 to 35.71% in the previous trial, compared to 46.7 to 26.4% in our trial, indicating a more effective reduction in the previous trial. This difference may be attributed to the selection of elderly patients more prone to hypovolemia in the previous study, and their use of a larger pre-rehydration volume of 8 mL/kg, compared to the 250 mL used in our trial.

Special attention should be given to choosing the categories of fluid therapy. Currently, colloids and crystalloids are available for selection. Colloids have better blood volume expansion and longer-lasting effective blood volume maintenance, including hydroxyethyl starch (HES), dextran, gelatin, and albumin (22, 23). They can reduce infusion volume, the incidence of pulmonary infection, and tissue edema (24). Therefore, colloids were preferred for fluid therapy in our trial. However, it’s important to note that excessive colloid administration carries potential risks such as kidney damage, coagulation abnormalities, and allergic reactions (25–30). Fortunately, these adverse reactions were not observed in our trial.

Our study has several limitations. First, we adopted FTc cutoff values directly from Chen et al. (13) trial without conducting specific experiments for patients with gastrointestinal tumors, potentially affecting the results. Second, FTc, as an ultrasound Doppler technology, requires significant learning time, which may pose challenges for clinical adoption. Lastly, we did not investigate the potential impact of hypotension during the induction period. Therefore, the clinical relevance of FTc in minimizing hypotension during this phase requires further validation through more clinical trials.

In conclusion, pre-rehydration with a cutoff value of FTc <340.7 ms can reduce the occurrence of hypotension induced by general anesthesia in patients with gastrointestinal tumors.

## Data availability statement

The original contributions presented in the study are included in the article/supplementary material, further inquiries can be directed to the corresponding authors.

## Ethics statement

The studies involving humans were approved by Medical Ethics Committee of the Cancer Hospital Affiliated with Chongqing University. The studies were conducted in accordance with the local legislation and institutional requirements. Written informed consent for participation in this study was provided by the participants’ legal guardians/next of kin.

## Author contributions

ML: Methodology, Writing – original draft. FL: Formal analysis, Software, Writing – original draft. JY: Data curation, Writing – original draft. XT: Formal analysis, Funding acquisition, Writing – original draft. CZ: Validation, Writing – original draft. QC: Conceptualization, Funding acquisition, Writing – review & editing. HL: Conceptualization, Supervision, Writing – review & editing.
